# The antibacterial efficacy of silver diamine fluoride (SDF) is not modulated by potassium iodide (KI) supplements: A study on *in-situ* plaque biofilms using viability real-time PCR with propidium monoazide

**DOI:** 10.1371/journal.pone.0241519

**Published:** 2020-11-03

**Authors:** Nizam Abdullah, Farah Al Marzooq, Suharni Mohamad, Normastura Abd Rahman, Koippallil Gopalakrishnan Aghila Rani, Hien Chi Ngo, Lakshman Perera Samaranayake

**Affiliations:** 1 College of Dental Medicine, University of Sharjah, Sharjah, UAE; 2 School of Dental Sciences, Universiti Sains Malaysia Health Campus, Kubang Kerian, Kelantan, Malaysia; 3 Department of Medical Microbiology and Immunology, College of Medicine and Health Sciences, United Arab Emirates University, Al Ain, UAE; 4 UWA Dental School, The University of Western Australia, Nedlands, Australia; 5 Faculty of Dentistry, University of Hong Kong, Hong Kong, China (SAR); University of the Pacific - Arthur A Dugoni School of Dentistry, UNITED STATES

## Abstract

Silver diamine fluoride (SDF) is commonly used to arrest caries lesions, especially in early childhood caries. Recently, it was suggested that SDF can be combined with potassium iodide (KI) to minimize the discoloration of demineralized dentine associated with SDF application. However, the antibacterial efficacy of SDF alone or combined with KI on *in-situ* biofilm is unknown. Hence, we compared the anti-plaque biofilm efficacy of two different commercially available SDF solutions, with or without KI, using an *in-situ* biofilm, analysed using viability real-time PCR with propidium monoazide (PMA). Appliance-borne *in-situ* biofilm samples (n = 90) were grown for a period of 6 h in five healthy subjects who repeated the experiment on three separate occasions, using a validated, novel, intraoral device. The relative anti-biofilm efficacy of two SDF formulations; 38.0% Topamine (SDF^T^) and 31.3%, Riva Star (SDF^R^), KI alone, and KI in combination with SDF^R^ (SDF^R+KI^) was compared. The experiments were performed by applying an optimized volume of the agents onto the biofilm for 1min, mimicking the standard clinical procedure. Afterwards the viability of the residual biofilm bacteria was quantified using viability real-time PCR with PMA, then the percentage of viable from total bacteria was calculated. Both SDF formulations (SDF^T^ and SDF^R^) exhibited potent antibacterial activities against the *in-situ* biofilm; however, there was non-significant difference in their efficacy. KI alone did not demonstrate any antibacterial effect, and there was non-significant difference in the antibacterial efficacy of SDF alone compared to SDF with KI, (SDF^T^ v SDF^R/KI^). Thus, we conclude that the antibacterial efficacy of SDF against plaque biofilms is not modulated by KI supplements. Viability real-time PCR with PMA was successfully used to analyze the viability of naturally grown oral biofilm; thus, the same method can be used to test the antimicrobial effect of other agents on oral biofilms in future research.

## Introduction

Dental caries, particularly in childhood, is a rampant, global health problem that affects millions [1]. The damage caused by dental caries is cumulative over time, it is therefore important to control the disease from early childhood [2]. A variety of evidence-based approaches for caries prevention strategies have been reported [3]. These include pharmacologic and chemotherapeutic modalities to simply arrest and halt the caries process [4–7]. One of these noteworthy approaches that has gained recent popularity is the topical application of silver diamine fluoride (SDF), particularly for early childhood caries (ECC) management [8,9]. Numerous clinical trials have demonstrated the effectiveness of SDF arresting both childhood and adult dental caries [1,4,10].

SDF was first used in Japan, in the late 1960s, for the specific purpose of caries management. However, until the beginning of the 21^st^ century it did not gain much popularity, due to its staining properties of the dental tissues subjacent to the carious lesion, and questions related to toxicity [11]. Nevertheless in 2014 SDF was finally approved by the US Food and Drug Administration authority as a class II medical device [12].

Currently, SDF is commercially produced either as a colorless, or as a blue tinted, highly alkaline aqueous solution containing fluoride, silver and ammonium ions. The precise role and mechanisms underlying the antibacterial activity of SDF is still unclear and rather speculative. Silver ions in SDF appear to have a trimodal action on bacteria. First, they appear to react with the bacterial cell wall components causing cell lysis and second, the ionic silver interferes with bacterial DNA synthesis and inhibit metabolic proteins required for cellular respiration, and finally, the ionic interactions of bacterial cellular surface proteins and silver ions are thought to prevent bacterial aggregation, a prerequisite for caries initiation and progress [13,14]. In addition, the silver ions react with hydroxyapatite of the tooth to form an impermeable black, outer layer on the carious tooth, arresting the lesion [15]. The foregoing properties of silver ions acting in tandem with bactericidal and remineralization activity of fluoride ions have been suggested as the major modes of SDF action, that lead to caries arrest, as shown in many clinical trials [15].

However, the major disadvantage of SDF, like other silver compounds, is the black staining effect of the carious tissues. This discoloration, caused by oxidation of ionized silver into metallic silver, is a major shortcoming, and limits its clinical use in the aesthetic zone particularly in demanding patients [15,16]. In 2002 Ngo et al. proposed that supplementation of SDF with potassium iodide (KI) as a solution to the problem of discoloration [17]. They noted that the application of a saturated solution of KI immediately after SDF application prevents the staining effects without altering the caries arresting effect of SDF. It has been suggested that the latter phenomenon is due to the reaction of silver ions, from the SDF solution, and iodide ions from KI forming a white precipitate of silver iodide [13].

Interestingly, the decreased staining of dentine along with qualitative reduction in biofilm formation following the application of KI alone has been reported in several, recent, *in vitro* studies [13,18]. Additionally, *in vivo* applications of either SDF or SDF plus KI have shown to inhibit the growth of the cariogen, *Streptococcus mutans* recovered from carious dentine samples [19]. Application of SDF plus KI is also reported to be effective in inhibiting development of secondary caries in glass ionomer cement (GIC) restorations [20].

It is difficult to discern from the foregoing clinical trials and *in vitro* studies, the relative bactericidal efficacy of SDF alone, and SDF plus KI combination. Hence the main aim of the current study was to evaluate the relative antimicrobial efficacy of two different commercially available SDF suspensions, namely, Topamine and Riva Star. SDF Topamine (SDF^T^) was tested alone while SDF Riva Star (SDF^R^) was tested alone and in combination with KI (SDF^R+KI^), and KI was tested alone. Sterile distilled water and 2% Chlorhexidine (CHX) were used as negative and positive controls, respectively. The experiments were done using *in-situ* biofilms, grown in five adult volunteers. For the latter purpose a recently developed and validated, intraoral device for *in-situ* biofilm growth was utilized [21]. To our knowledge, this is the first study to report the relative antimicrobial efficacy of SDF and KI using standardized, *in-situ* biofilms from adult subjects.

## Materials and methods

### Antimicrobial agents

The relative anti-plaque biofilm efficacy of the following agents were evaluated; SDF Topamine (38% w/v) alone -SDF^T^; SDF Riva Star (31.3% w/w)- SDF^R^; SDF Riva Star+ KI—SDF^R+KI^, KI alone, sterile distilled water as negative control, and 2% CHX as positive control (Table 1). Riva Star is the only product available as SDF and KI in separate single dose vials, for sequential application in clinical settings.

**Table 1:** Agents used in the study.

^#^ Agents	Batch	Manufacturer	Application method
SDF^T^	PD160.9–1	PharmaDesign Co.,Ltd, Samutprakam, Thailand.	*2μl solution was passively applied into the biofilm well for one minute
SDF^R^	8800505	SDI, Bayswater, Australia	Same as *
SDF^R+KI^	8800505	SDI, Bayswater, Australia	2μl solution from the silver capsule was passively applied into the biofilm well, immediately followed by 2μl solution from the green capsule for one minute.
KI	8800505	SDI, Bayswater, Australia	Same as *
Chlorhexidine 2%	1111906	Prevest DenPro Limited, Jammu, India	Same as *
Sterile distilled water (laboratory grade)	W4502	Sigma, Dorset, United Kingdom	Same as *

^#^ SDF^T^: SDF Topamine, SDF^R^: SDF Riva Star, SDF^R+KI^: SDF Riva Star+KI.

### Study subjects

Five volunteers, 2 males and 3 females, age 39±4.32 years, all dental staff working in the College of Dental Medicine, University of Sharjah, United Arab Emirates were recruited into the study on 2^nd^ December 2019. Inclusion criteria were healthy non-smokers with no active caries, having activated salivary flow rate of at least 1ml/min, and subjects should not have been on antibiotics within the last two weeks and does not use daily mouth rinses. Exclusion criteria were those with systemic diseases, wearing removable prosthesis or orthodontic appliances, on antibiotics, using oral antiseptic in the past three months, or on medications that may influence production or composition of saliva.

A single investigator (first author) collected all the samples to obviate any inter investigator variation.

Ethical clearance was obtained from the Ethics Committee of the University of Sharjah (REC-17-10-08-01-S dated 26/11/2017) and Universiti Sains Malaysia (USM/JEPeM/19030208). Written informed consent was obtained from each subject before commencement of the study and confidentiality was protected by rendering the samples anonymous.

### Growth and collection of the *in-situ* biofilm

**Fig 1:**
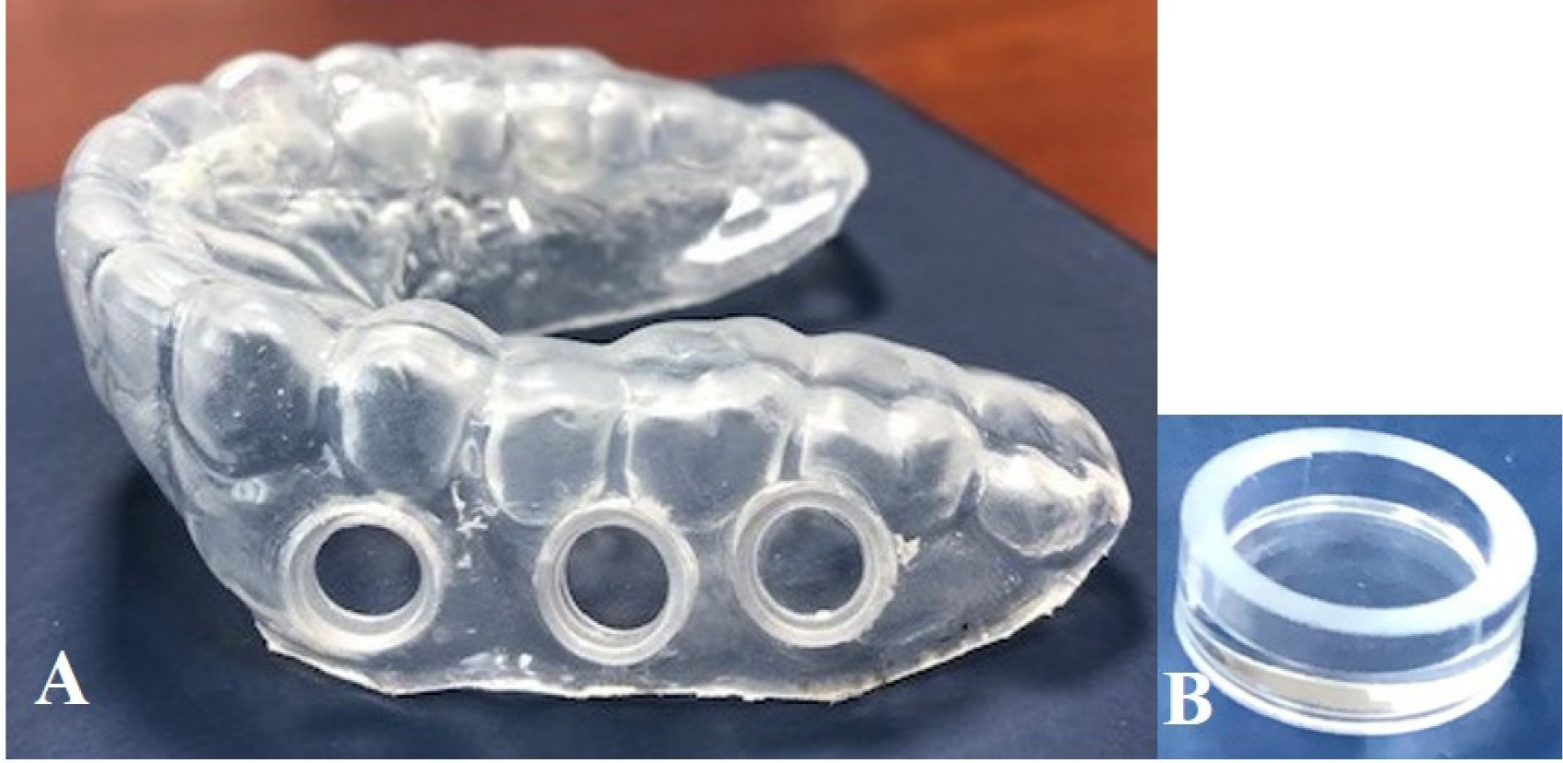
The intraoral device for *in-situ* biofilm collection. A: Intraoral device with removable wells, showing three different pits where the removable wells are inserted; B: An image of a removable well submerged within the dimensionally fitting well in the device.

All subjects who participated in the study underwent a professional oral hygiene prophylaxis on the day before the start of the clinical study. They then wore individualized acrylic intraoral device on the upper jaw for a continuous, period of 6 h for collection of oral biofilms. The device carried six cylindrical, removable “wells”, measuring 5 mm diameter x 3 mm height, three on each side of the jaw, for biofilm growth (Fig 1). The device was worn continuously, except during meal- times where it was stored and kept moist. Subjects were allowed to rinse the mouth lightly with water, only after meals. The clinical experiment took place from 16^th^ December 2019 until 3^rd^ February 2020.

### Optimization study: Determining the lowest inhibitory volume of SDF on the *in-situ* biofilm

Four subjects took part in this experiment in December 2019. Each of them wore an individualized acrylic intraoral device on the upper jaw carrying six cylindrical removable “wells” for biofilm growth. At the end of the 6 h period, the device was immediately transported to the laboratory, the “wells” were removed from the device and the biofilms from five wells were each treated with different volumes of SDF Topamine; 0.5, 1.0, 2.0, 5.0 and 10 μl using a micropipette. The sixth well was free from SDF treatment for use as a negative control.

Following exposure to the agent for 1min, the biofilms were extracted from each well by brushing the base of the well (10 times clockwise), using a pre-sterilized regular size micro-brush (MD Medical, USA). The biofilm attached to the micro-brushes was then suspended in 600 μl Brain-Heart Infusion (BHI) broth for microbial processing and analysis to determine the lowest inhibitory volume of SDF.

### Experiments to evaluate the antibacterial efficacy of SDF with and without KI using *in-situ* biofilms

Five subjects took part in this experiment that began in January 2020. They wore an individualized acrylic intraoral device on the upper jaw carrying six cylindrical removable “wells” for biofilm growth. At the end of 6 h, the “wells” were removed from the device. Based on results of optimization study, 2 μl of the six test agents (Table 1); SDF^T^, SDF^R^, SDF ^R+KI^, KI, sterile distilled water and CHX were applied onto each of six separate biofilms within wells, using a micropipette, and were exposed to the agents, and the controls for 1min. Afterwards, the biofilms from each well were harvested, into separate vials, using a pre-sterilized micro-brush as described above.

In the SDF ^R+KI^ test well, SDF was applied first and immediately followed by KI as per the clinical application process (Table 1), the mixture was left for 1min, after which the biofilms were collected as described above. Each subject repeated the experiments on three separate occasions with two weeks rest period in between from 6^th^ January to 3^rd^ February 2020).

### Microbial processing and quantification of viable and total bacteria

The biofilms were processed further for live/dead bacterial staining and analysis using viability real-time PCR kit with PMAxx dye (Biotium, Fremont,CA, USA), a high affinity photoreactive DNA binding dye which can bind to DNA of non-viable bacteria with compromised cell membrane [22]. Briefly, 600 μl of each biofilm suspended in BHI broth was equally aliquoted into two vials (300 μl each) and analyzed for total and viable bacterial counts. To determine viable bacteria, the biofilms were stained with PMAxx dye according to the manufacturer’s instructions.

Briefly, working in dim light, 0.6 μl PMAxx dye was added to 300 μl samples, covered immediately with aluminium foil and incubated for 10- minutes. After incubation, the aluminium foil was removed and samples were exposed to blue light using a Glo-Plate^TM^ Blue (Biotium, Fremont,CA, USA) for 15- minutes. This photoactivation step is essential for the dye to covalently bind to the dead cell DNA.

Total genomic DNA was isolated from both stained and unstained biofilm samples using a modified protocol of Epicentre MasterPure^TM^ DNA Purification Kit (Epicenter, USA). DNA quality was assessed by spectrophotometry and the samples used had a nucleotide:protein ratio (260:280) within the range of 1.8 and 2.0.

### Quantification of live/dead bacteria using viability PCR

Bacterial 16S rRNA gene was amplified using real time PCR in StepOne^TM^ Real-Time PCR System (Applied biosystems, USA). Amplification was performed in a total reaction volume of 20 μl containing 5x HOT FIREPol^®^ EvaGreen^®^ qPCR Mix Plus (Solis BioDyne, Estonia) and optimized concentration (0.4 μM) of primers manufactured by Macrogen, Korea. The primers used were universal primers for 16S rRNA gene: forward primer 5’-TCC TAC GGG AGG CAG CAG T-3’ and reverse primer 5’-GGA CTA CCA GGG TAT CTA ATC CTG TT-3’ with the annealing temperature of 60°C. PCR amplification of dye-modified DNA templates (dead bacteria) is inhibited, allowing selective quantitation of DNA from viable cells. For each biofilm collected, PCR amplification was performed for the dye treated sample (to determine the viable count) and also for the non-treated sample (to determine the total count composed of both viable and dead bacteria). All the PCR amplification reactions were performed in duplicate.

A critical threshold cycle (Ct) value was derived from the foregoing viability real-time PCR assay, and the Ct value was converted to colony-forming unit (CFU/mL) with the help of a standard curve. The calibration curve was obtained by using purified genomic DNA extracted from a suspension of *S*. *aureus* (ATCC 25923) with optical density equivalent to 0.5 McFarland standard [containing ~10^8^ bacterial colonies (CFU/ml)]. A ten-fold serial dilution was then performed starting from the concentration of 10^8^ CFU/ml to yield concentrations ranging from 10^7^ to 10^1^ CFU/ml. All dilutions were performed in duplicates. Calibration curves and conversion from Ct to CFU/ml were done using GraphPad Prism^®^ Version 8.0 (GraphPad Software, USA).

### Statistical analysis

SPSS software version 22 (SPSS Inc., Chicago, IL, USA) was used to perform all statistical analyses. Data were expressed as mean ±SD. One-way ANOVA test was used to compare percentage of viable bacteria between groups. P < 0.05 was considered statistically significant.

## Results

### Antibacterial effect of different volumes (concentrations) of SDF (Topamine) on *in-situ* biofilm using viability real-time PCR

**Fig 2:**
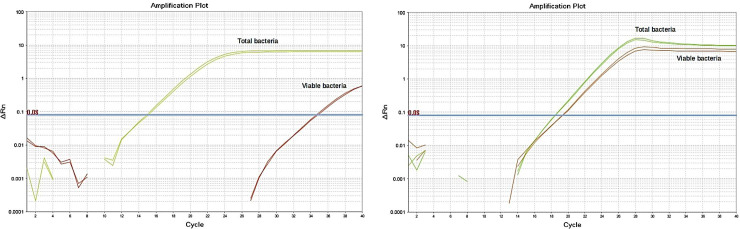
Amplification plots of biofilm samples treated with SDF (A), compared to untreated control (B). Green curves represent amplification plots from biofilm samples not treated with PMAxx dye (amplification from total bacteria present in the sample) and the red curves represent amplification plots from biofilm samples treated with PMAxx dye (amplification from viable bacteria, excluding dead bacteria). Each sample was tested in duplicate (as shown in the figure).

Cumulatively, a total of 24 *in-situ* biofilm samples were collected for this experiment. To determine the lowest inhibitory volume of SDF^T^ that significantly inhibits the plaque biofilm, each of a serial dilution (0.5, 1, 2, 5 and 10μl) of SDF^T^ and negative control were applied onto 4 *in-situ* biofilm samples. Viability real-time PCR was used to estimate the amount of viable bacteria after treatment with SDF (Fig 2).

**Fig 3:**
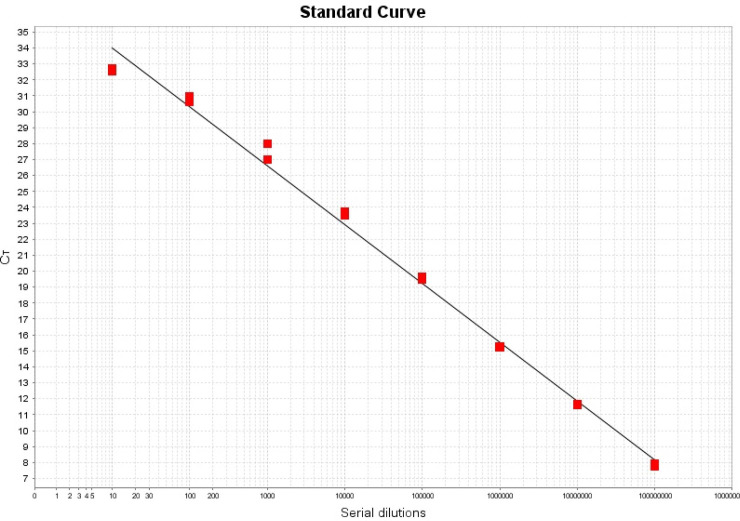
Calibration curve (standard curve) used for the conversion of Ct values to the equivalent bacterial concentration in CFU/ml. A serial dilution of Staphylococcus aureus (108 to 10 CFU/ml) was used as a standard for the generation of the calibration curve.

Fig 2A demonstrates the amplification from *in-situ* biofilm treated with SDF, compared to non-treated biofilm shown in Fig 2B. Biofilm treated with PMAxx dye is shown in the (Fig 2A and 2B), and it represents amplification from viable bacteria (red curves), while total bacteria including both viable and dead bacteria were amplified from samples not treated with PMAxx (green curves). A standard curve, exemplified in Fig 3 was used to convert the Ct values to concentrations in CFU/ml, then the percentage of viable from total bacteria was calculated.

**Fig 4:**
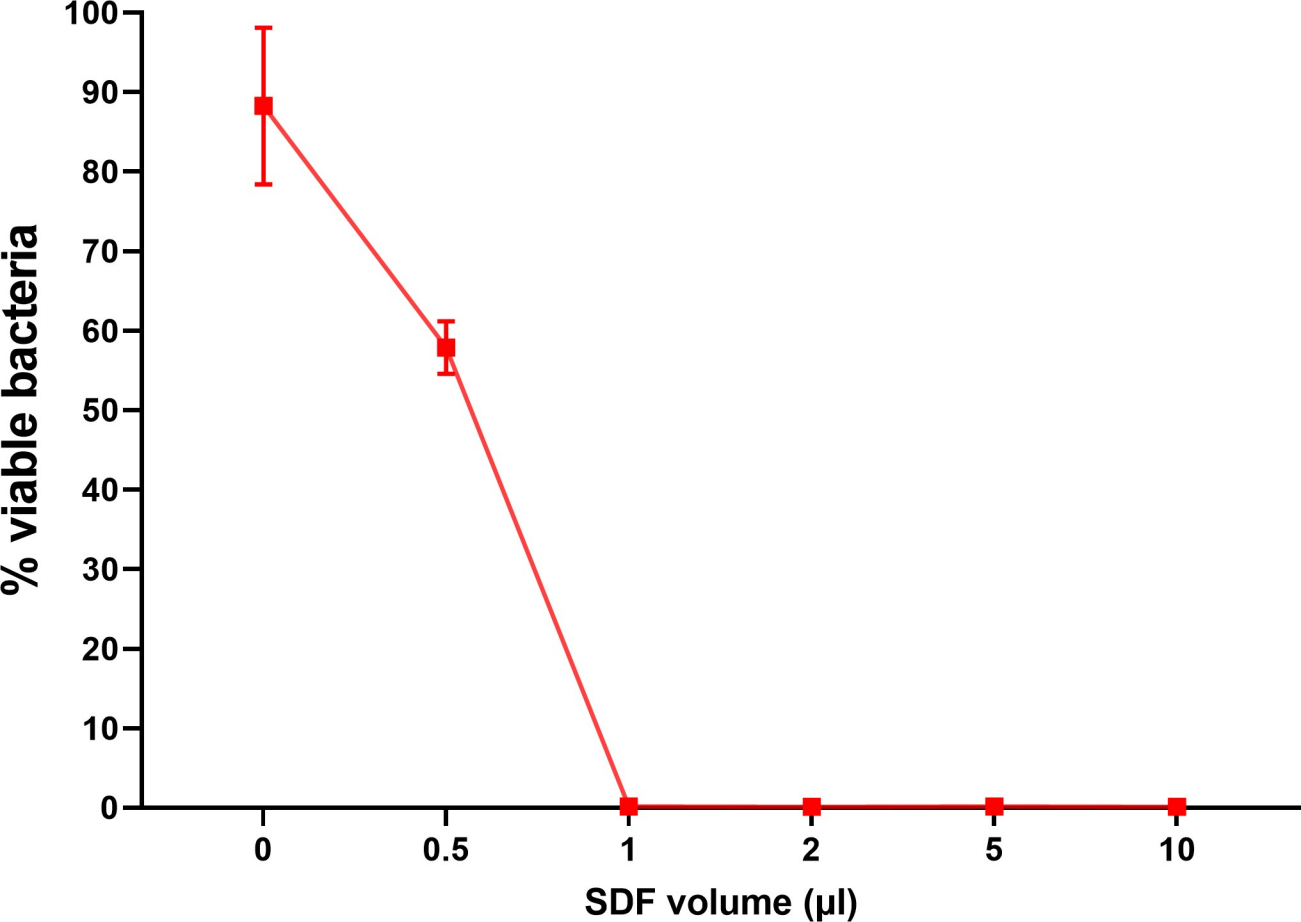
The antibacterial effect of different volumes of SDF applied on *in-situ* biofilm (n = 4 for each volume). Percentage of viable bacterial residue after treatment are shown. For 1–10 μl of SDF, mean % viable bacteria were less than 0.25%. The data shown was derived from samples collected from four subjects (mean ± SD of % viable bacteria).

As shown in Fig 4, there was a significant reduction (p<0.05) in percentage of viable bacteria in all the test samples compared with the negative control (volume 0 shown in Fig 4). Treatment with 0.5 μl of Topamine (38%) caused about 30% reduction in the % of viable bacteria compared to the non-treated biofilm [as shown in Fig 4 (0.5 compared to 0)]. Biofilms exposed to 1, 2, 5 and 10 μl volumes of SDF showed significant reduction in viable bacteria compared to biofilms exposed to 0.5 μl (p<0.05). However, there was no significant difference observed between 1, 2, 5 and 10 μl of SDF exposed biofilms (p>0.05), and hence the lowest biofilm inhibitory volume of SDF of 2 μl was chosen for use in the subsequent experiment as the volume is similar to that carried by a fine micro-brush used in clinical application.

### Antibacterial efficacy of SDF^T^, SDF^R^ and SDF ^R+KI^

**Fig 5:**
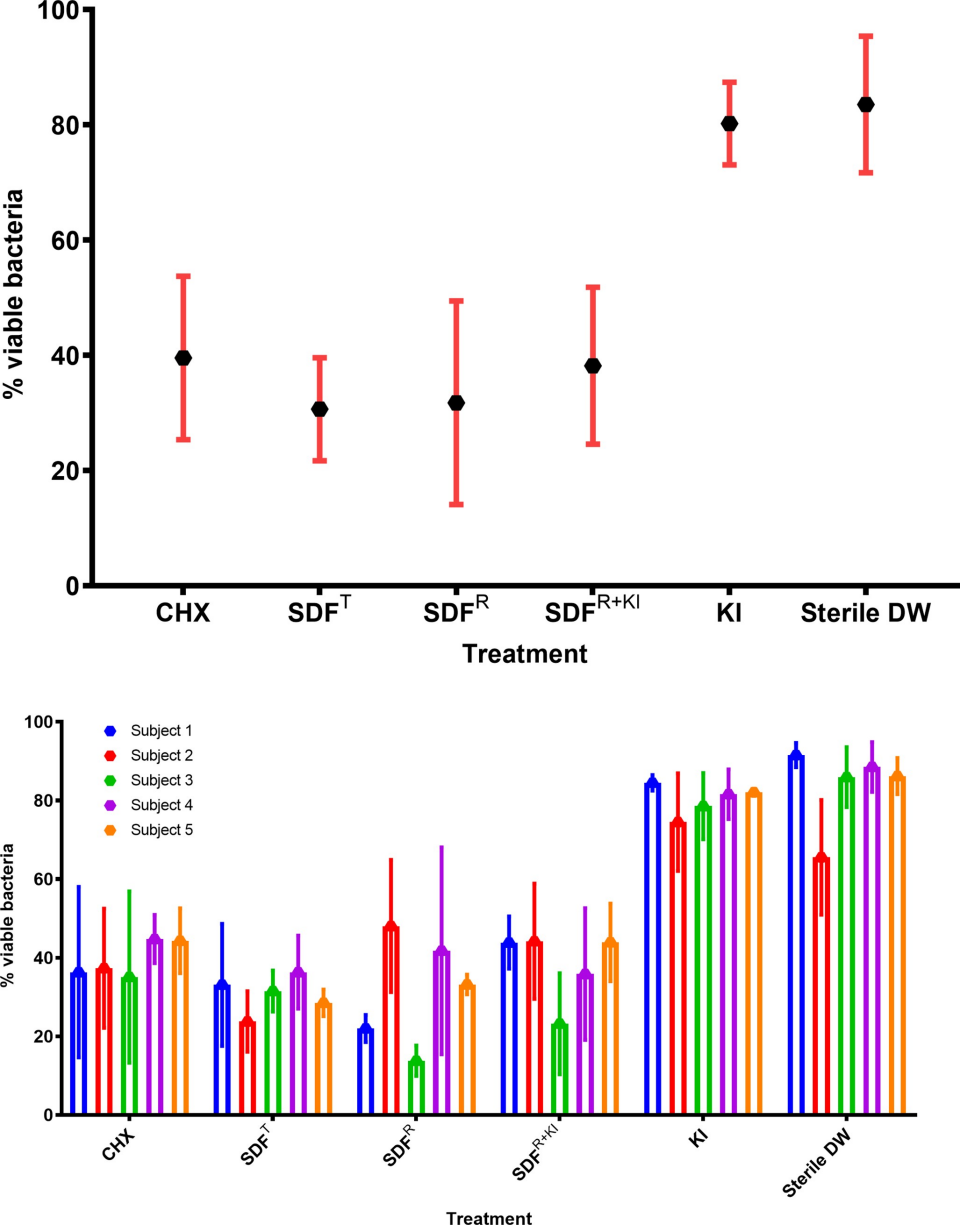
A. Percentage of viable bacteria (mean ± SD) in *in-situ* biofilms after treatment with different antibacterial agents (CHX: 2% Chlorhexidine- as a positive control, SDF^T^: SDF Topamine, SDF^R^: SDF Riva Star, SDF^R+KI^: SDF Riva Star + Potassium Iodide, KI: Potassium Iodide, DW: Distilled Water- as a negative control), n = 15 for each agent. B. Percentage of viable bacteria (mean ± SD) in *in-situ* biofilms after treatment with different antibacterial agents (CHX: 2% Chlorhexidine- as a positive control, SDF^T^: SDF Topamine, SDF^R^: SDF Riva Star, SDF^R+KI^: SDF Riva Star + Potassium Iodide, KI: Potassium Iodide, DW: Distilled Water- as a negative control), for each subject.

A total of 15 *in-situ* biofilm samples were collected for treatment with each test agent, and we analysed, cumulatively, 90 samples from all five subjects. On analyses, of the residual viable biofilm bacteria between SDF^T^, SDF^R^, SDF ^R+KI^ and the positive control, CHX, no significant difference was noted between the test and control samples implying that KI supplementation of SDF had no additional antibacterial effect (p> 0.05; Fig 5).

**Fig 6:**
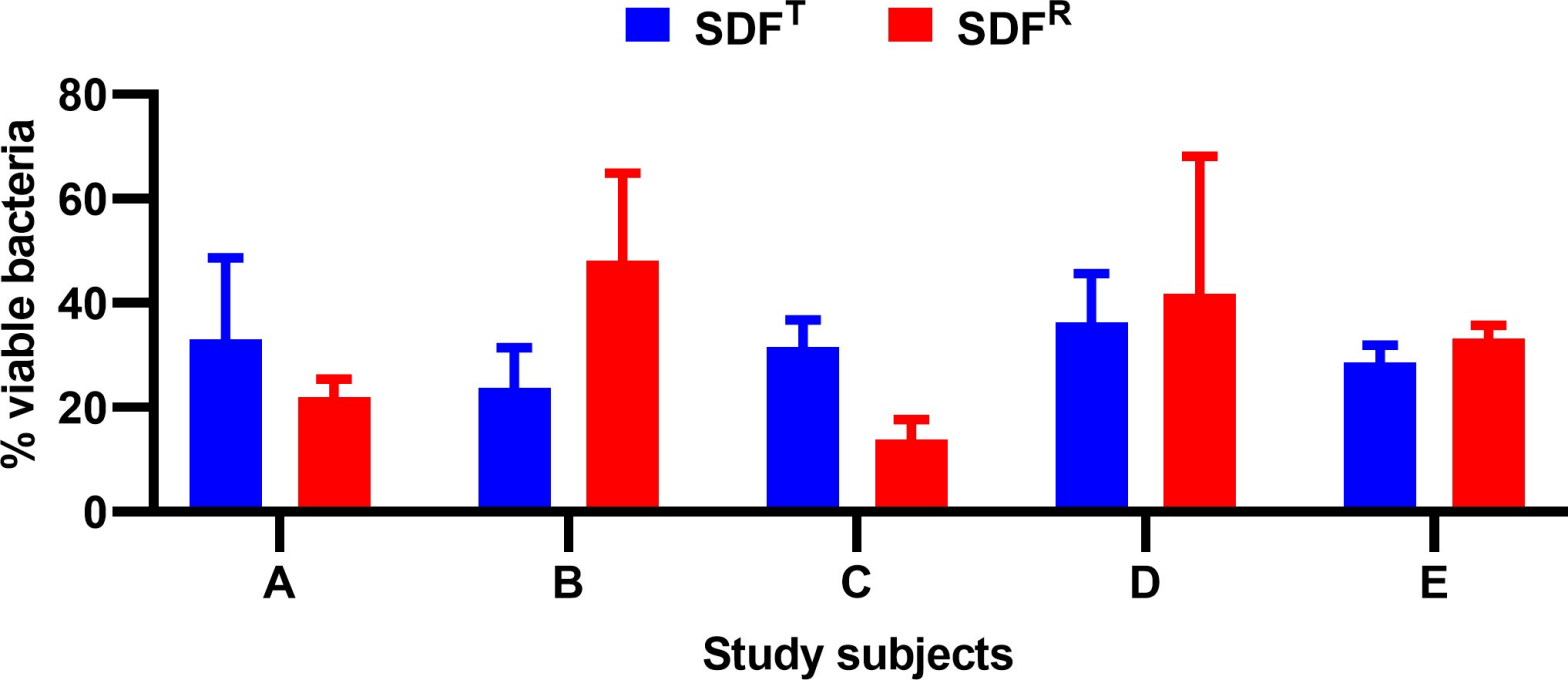
Percentage of viable bacteria upon treatment with SDF^T^ (Topamine) and SDF^R^ (Riva Star) among the study subjects. Wilcoxon signed rank test was used and the difference was not significant (p>0.05).

The percentage of the residual viable biofilm plaque bacteria after SDF^T^, SDF^R^, SDF ^R+KI^ and CHX application were 30.64 (± 8.95), 31.75 (± 17.67), 38.19 (±13.62), and 39.55 (±14.18), respectively. KI alone, in comparison to distilled water controls, did not exert any antibacterial effect on plaque biofilms (p >0.05; Fig 5). Furthermore, there was no significant difference in the anti-biofilm efficacy of Topamine (SDF^T^,) and Rivastar (SDF^R^) with two different concentrations of SDF, 38% and 31.3%, respectively (p >0.05; Fig 6).

## Discussion

Dental caries, a complex biofilm-mediated disease, is still a major health problem in most industrialized countries affecting both children and adults. Better knowledge on the characteristics of oral microbiome results in the development of better management strategies focusing on proactive management of oral health through an ecological approach to its symbiotic residents. Our therapeutic goal is to re-establish symbiosis equilibrium of oral microorganism by modulation of oral biofilms using various antiplaque agents.

We investigated for the first time, using natural plaque, the bactericidal effects of SDF (Topamine; SDF^T^), SDF (Riva Star; SDF^R^), SDF+KI (Riva Star; SDF ^R+KI^) and KI alone on 6 h *in-situ* biofilms, using a validated, novel biofilm device. The key finding of the study is that application of KI following SDF application (SDF ^R+KI^), a standard clinical practice, does not significantly reduce its bactericidal efficacy when compared to SDF solutions (SDF^T^ or SDF^R^) applied alone.

To our knowledge, this is the first study comparing the antibacterial efficacy of SDF and SDF+KI on *in-situ* biofilms. Previous studies utilized *in vitro* methods by growing mono-species biofilms [23], or dual/multispecies biofilms of *Streptococcus mutans*, *Actinomyces naeslundii*, *Lactobacillus acidophilus*, by growing them on dentine blocks [5,24]. The latter studies revealed that SDF significantly inhibited mono or/and dual, multispecies biofilms.

No doubt the creation of *in vitro* biofilm models has contributed to significant advances in the study of oral diseases [25–27]. However, their greatest disadvantage is the inability to mimic a complex plaque eco-system comprising both cultivable and an uncultivable organisms of a multi species biofilm comparable to those found *in-situ* [28–31]. Yet, the *in vitro* grown biofilm models enable investigators seek the outcome of biofilm growth under standardized and simplified conditions to resolve defined biological issues. Additionally, under controlled laboratory conditions, the *in vitro* studies are relatively easier to conduct and reproduce. This is in contrast to the *in vivo* and *in-situ* experiments that mimic natural oral conditions which are inherently more complex but yield perhaps more realistic outcomes [21]. Hence, in this study we used a validated, biofilm generating device which could be worn by volunteers to grow plaque biofilms *in-situ* under standardized conditions.

The major drawback to SDF treatment has been the black residual stain, which has limited its use particularly in the aesthetic region of the dentition. This has been a major concern for patients from the pediatric population as well as their parents, especially in the case of ECC management [11,32]. Therefore, to overcome the staining phenomenon, and to increase patient acceptance, application of KI immediately after SDF application has been suggested [17]. It has been theorized that KI prevents staining through precipitation of excess silver ions as a white silver iodide deposit [33]. On the contrary, however, although a number of *in vitro* studies have clearly shown that treatment of teeth with KI, after SDF application reduces tooth discoloration [13,33,34], a very recent systematic review has reported that the weight of current evidence fails to support the role of KI in the management of SDF-associated tooth discoloration [35]. Hence, further work is clearly required to resolve this dispute.

One group of workers have determined that SDF inhibits bacteria in a dose dependent manner [2]. Hence the volume of SDF delivered to the oral biofilm is a critical factor in determining its antibacterial efficacy on *in-situ* biofilms. We, therefore, standardized this particular variable in our study by conducting an adjunct experiment, to simulate the volume of SDF delivered to an *in-situ* biofilm using a micro-brush as used in general clinical practice. Our results showed that 2 μl of SDF, a volume similar to that carried by a fine micro-brush (unpublished data) to be the optimum efficacious dose for evaluating the anti-bacterial effect on a 6 h *in-situ* biofilm. Therefore, 2 μl was chosen as the standard delivery volume of SDF throughout our study.

The absence of a significant biofilm antibacterial efficacy of two different commercially available solutions of SDF alone, compared to SDF+KI combination is in concordance with an *in vitro* study which revealed that the effectiveness of SDF in caries arrest was not affected or minimally affected by the application of KI [34]. Indeed, another *in vivo* study utilizing *S*. *mutans* from human dentinal caries samples also did not show significant difference between SDF alone and SDF+KI combination [19].

Currently, SDF is available in various countries in concentration ranging from 10% to 38%, with 38% being the commonly used concentration for the management of tooth hypersensitivity and caries control [35]. Nevertheless, there are very few studies comparing the anti-plaque biofilm activity of differing concentration of SDF. In a previous *in vitro* study, De Almeida et al [2] found a dose dependent antibacterial activity on comparing 12% and 30% using a commercially available SDF suspension (Cariestop). On the contrary, we did not observe a significant dose dependent activity of SDF solutions against the plaque biofilm despite the two different concentrations of the chemicals (Topamine-38% and Riva Star 31.3%).

Chlorhexidine gluconate (CHX), the gold standard antimicrobial agent commonly used as a cavity cleanser [36,37], was used as a positive control in this study. Our results, based on viability PCR, showed that bactericidal activity of both SDF solutions alone and SDF+KI was not similar to that of CHX. Recently, Karched et al., (2019) evaluated the *in vivo* effect of SDF and SDF+KI on dentine caries lesions, using standard culture methods, also reported similar data [19]. Nevertheless, there are two previous *in vitro* studies where SDF (Riva Star) was noted to be more effective as an antibacterial agent than 2% CHX [13,38]. Hence further work, both *in vivo* and *in vitro*, are required to shed light on this controversy.

To our knowledge, this is the first study analyzing live/dead *in-situ* plaque bacteria using the method of Viability real-time PCR and the aid of propidium monoazide stain. We found this method to be reproducible, sensitive, simple and rapid, as previous workers [39–41]. On the other hand, various other microbiological techniques ranging from Colony Forming Units (CFU) quantification on culture plates [5,19,23,24] or visualizing under Confocal Laser Scanning Microscopy (CLSM) with fluorescent LIVE/DEAD stain [5,13,42] have been used by previous investigators for the same purpose. These methods have many limitations such as inability to detect unculturable bacteria by standard microbiology culture methods and difficulty in quantifying live bacteria using CLSM. We wish to commend the viability real-time PCR method for future workers in this field to generate comparable data, due to its afore mentioned properties which make it superior to other traditional techniques.

## Conclusion

To conclude, our data indicate, SDF and KI in combination has no significant advantages in terms of *in-situ* anti-plaque biofilm activity, compared to SDF alone. While KI alone does not exhibit any antibacterial activity against *in-situ* biofilms, its main advantage appears to be the better aesthetic outcome when used in combination. Finally, it is likely that, different clinical concentrations of SDF, used widely, has no dose response effect against plaque biofilm activity, although further work is required to confirm or refute our findings reported here.
